# Supplementary material for: Relative Risk of Death in Bulgarian Cancer Patients during the Initial Waves of the COVID-19 Pandemic
Source: Healthcare (Basel). 2023 Sep 20;11(18):2594. doi: 10.3390/healthcare11182594 (PMC10531457; doi:10.3390/healthcare11182594)

Figure S1. Quantile–quantile (Q–Q) plot of the residuals from the linear regression model of relative risk of death for all cancer patients group. The *p*-value = 0.4125 is from the Shapiro-Wilk test suggesting no evidence to reject the null hypothesis for normal distribution of the error terms in the model.

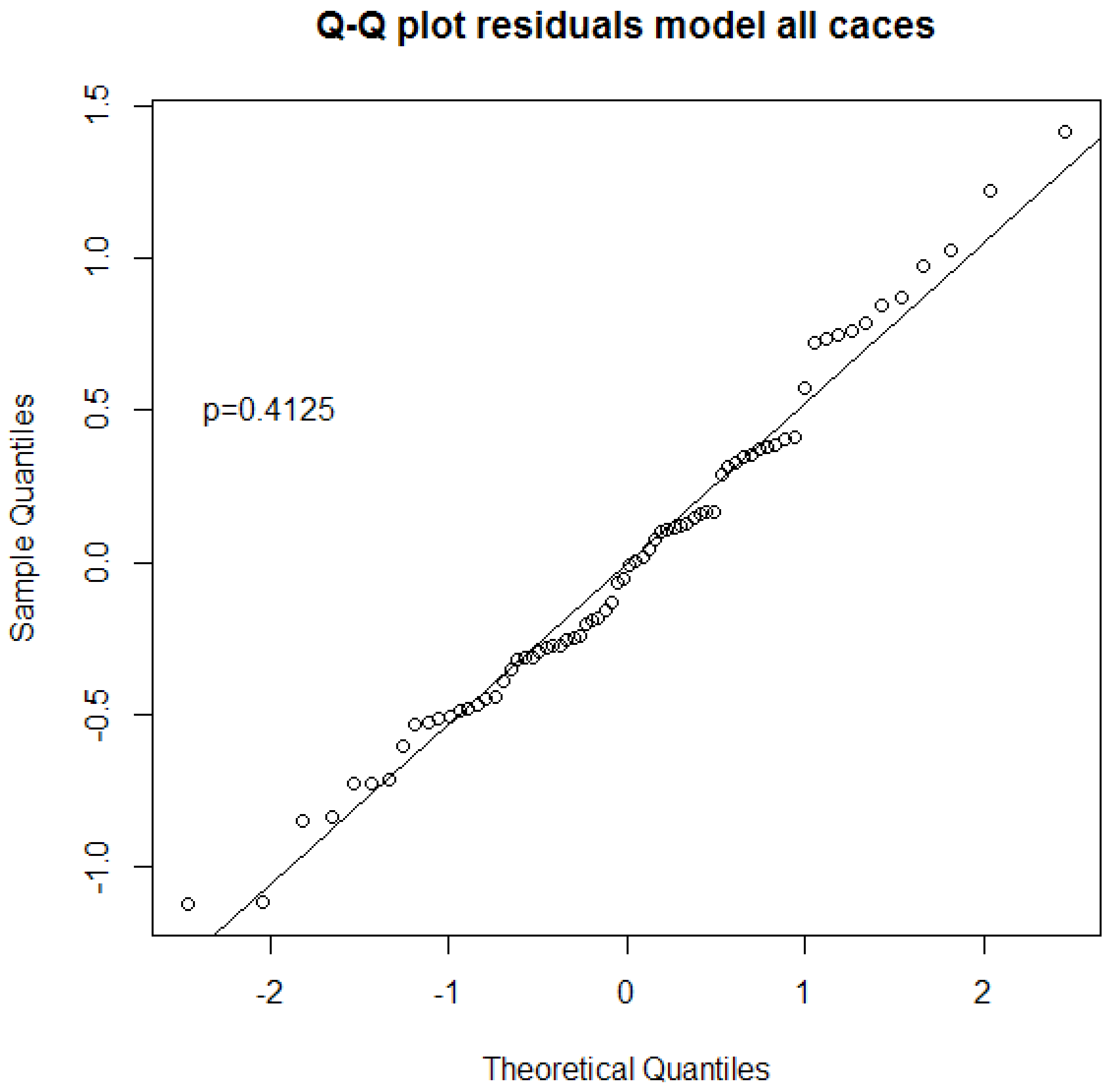

Supplement: Supplementary file 1 [file healthcare-11-02594-s001.zip › healthcare-2605323-supplementary.pdf]
